# Q & A with *FASEB BioAdvances* Editors‐in‐Chief

**DOI:** 10.1096/fba.2022-00064

**Published:** 2022-06-24

**Authors:** Crislyn D'Souza‐Schorey, Yung Hou Wong, Jeannine Botos, Darla P. Henderson

**Affiliations:** ^1^ University of Notre Dame Notre Dame Indiana USA; ^2^ Hong Kong University of Science and Technology Hong Kong China; ^3^ Department of Publications Federation of American Societies for Experimental Biology Rockville Maryland USA

## 1 TELL US A LITTLE ABOUT YOURSELF

**FIGURE 1:**
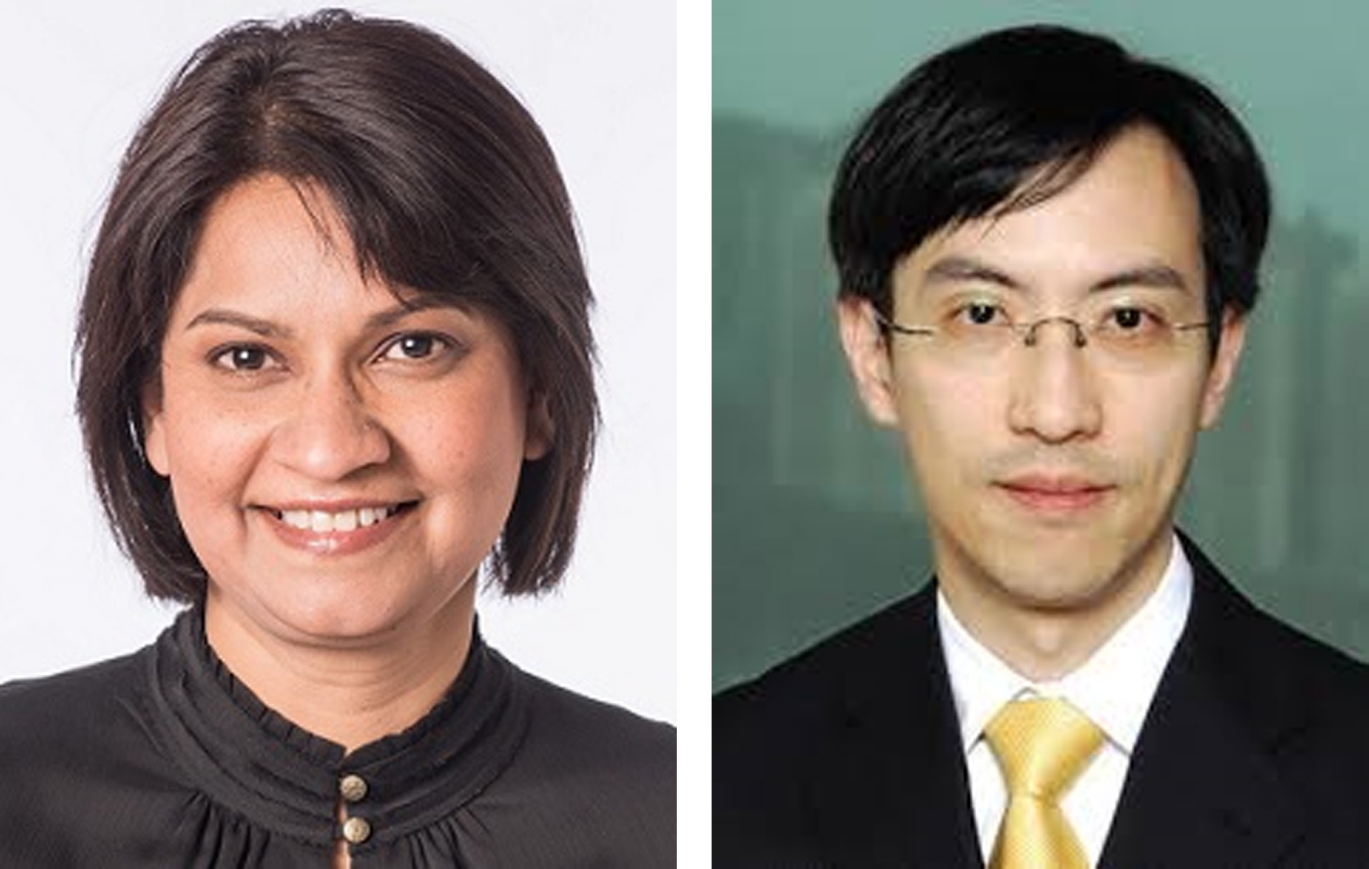
*FASEB BioAdvances* Editors‐in‐Chief Crislyn D'Souza‐Schorey (left) and Yung Hou Wong (right).

Yung (Figure 1, right): I received my doctoral degree in Pharmacology from the University of Cambridge and conducted postdoctoral training at the University of California San Francisco. Since the early 1990s, I have established my own research laboratory at the Hong Kong University of Science and Technology. As a molecular pharmacologist, my research interests are primarily focused on the delineation of signaling mechanisms regulated by the G protein‐coupled receptors (GPCRs). I have examined the G protein‐coupling specificities and effector systems of GPCRs for opioid peptides, melatonin, and chemokines, and explored how these and other GPCRs regulate gene transcription and cell proliferation and differentiation.

Crislyn (Figure 1, left): My research interests lie at the intersection of two exciting areas of biomedical research—Cell Biology and Molecular Oncology. We examine the cellular changes that accompany the early stages of tumor progression. A current line of investigation in my laboratory is to understand the biogenesis and paracrine properties of extracellular vesicles in the tumor microenvironment. For my doctoral studies and then postdoctoral research, I had the privilege of training and working with two outstanding scientists, both statesmen in their fields. Their labs provided environments that not only consolidated my enthusiasm for research but also tendered a vision for mentoring and leading. I joined the faculty at the University of Notre Dame in the late 1990s, where I established my independent research program.

## 2 WHAT MADE YOU MOST INTERESTED IN THE ROLE OF *FASEB BIOADVANCES* EDITOR‐IN‐CHIEF?

Crislyn: The FASEB organization is well recognized in the biological and biomedical science communities as the largest US biomedical coalition, representing 28 societies and well over 100,000 researchers. I recollect learning about FASEB's high repute as a graduate student. So, in 2020, I gladly accepted the invitation to serve as Associate Editor of their newly launched open‐access journal, *FASEB BioAdvances*. I am an advocate of open‐access publishing and the broad and inclusive reach it engenders. Open‐access publishing can transform scientific discourse by taking full advantage of the online platform. I was honored to be nominated and the selected as Editor‐in‐Chief by the search committee. It is a tremendous opportunity to serve the broader scientific community.

Yung: Modern‐day science has become increasingly multidisciplinary in nature, and this is especially true in biological and biomedical research. Seminal discoveries through interdisciplinary research will undoubtedly be needed to address complex biomedical challenges of the 21st century. *FASEB BioAdvances* provides a unique publication platform for the rapid dissemination of high‐quality research papers with a wide coverage. As an Editor‐in‐Chief of *FASEB BioAdvances*, I will have the privilege to read exciting scientific discoveries and advancements contributed by colleagues around the world. Additionally, I will have a great opportunity to work closely with a superb team of editors and editorial office staff, with a common goal of elevating the quality and impact of the journal within the biomedical community.

## 3 WHAT IS YOUR VISION FOR *FASEB BIOADVANCES* ?

A major motivation and reward for us as editors would be to see the journal grow with increasing quality and impact and have a global reach. We would like to see *FASEB BioAdvances* become a trusted venue for open‐access publishing in the biological and biomedical sciences. We are hoping to develop a journal editorial structure that is as diverse as the research community it represents, both scientifically and demographically. Over the past decade or so, we have seen a shift in the overall approach to life science innovation and discovery. This shift is driven by the need to address real‐world challenges, solutions to which require us to cut across siloed disciplines, transform existing disciplines, and generate new ones. The scope of *FASEB BioAdvances* provides an online platform for the dissemination of scholarship at both the frontiers of life science disciplines and also those that cut across boundaries. We will add that since FASEB is a recognized collective policy voice of biological and biomedical researchers, so we will continue to publish perspectives and opinion pieces on the broader issues affecting academia and biomedical education, science, and society.

## 4 WHAT SHOULD THE COMMUNITY KNOW ABOUT YOU AND THE *FASEB BIOADVANCES* EDITORIAL TEAM AND STAFF?

Both of us had previously served as Associate Editors of *FASEB BioAdvances* and thus have an inherent sense of affiliation with the journal. We are extremely proud to be working in tandem with an outstanding group of scientists on our board. Our editorial team reflects a balanced global representation of the leadership in biological and biomedical science. Our editorial staff are highly experienced and committed to the success of this promising journal. We also enjoy a strong partnership with *The FASEB Journal*. Together, we aim to have a global impact by publishing the highest‐quality research from researchers around the world, while working hard to embrace the value of diversity and inclusion at all levels of the journal.

## 5 WHAT ARE THE ADVANTAGES OF TWO EDITORS‐IN‐CHIEF, AND HOW WILL YOU WORK TOGETHER?

Given our prior Associate Editor appointments, we both have insights into the workings of the journal. As EICs, it has been both helpful and enjoyable to be able to share and bounce ideas off each other as we strategically plan for the future. We are located in different parts of the world, so in addition to our shared interests as scientists, we each bring unique perspectives to the table. We have regular virtual meetings, often alongside the Director of Publications, and have developed a working relationship that ensures seamless management and sharing of responsibilities.

## 6 WHAT CHANGES HAVE YOU MADE TO THE JOURNAL SINCE STARTING IN JANUARY?

We recognize the importance authors place on high‐quality and unbiased peer review conducted in a timely manner. In the next development phase of *FASEB BioAdvances*, we aim to provide authors with the highest standard of manuscript review, editing, and publishing, as well as to expedite the processing of submitted manuscripts. We have started to revamp our website so as to facilitate navigation by readers and authors. As mentioned earlier, we recognize the value of ensuring that our journal represents the diverse biomedical research being conducted across the globe. Hence, we are expanding the composition of our Editorial Board and Associate Editors to encompass broader expertise and geographical representation. We expect that all these changes will appeal to a broader range of readers.

## 7 WHAT CHALLENGES AND BOTTLENECKS DO THE RESEARCH AND PUBLISHING COMMUNITIES FACE TODAY?

There are several, but we will mention only a few here. The global research community is facing tremendous pressure to publish, as scientific publications have become the primary evaluation criteria for academic promotions and career advancement. Academic publishing, which relies on robust peer review at its core, is at risk with the alarming rise in sham or predatory platforms. Shifting standards of research communication, metrics, and rankings have created more space for predatory academic practices to take root and even flourish. Nowadays, impactful biomedical research often requires the amalgamation of diverse expertise to address complex biological questions, a feat that not every established researcher can attain with ease, let alone those who are at the early stages of their academic careers. Concurrently, the increasing level of sophistication in cutting‐edge high‐end instrumentation and their costs—for example, cryo‐electron microscopes—present a separate set of challenges in many parts of the world. To address these issues, collaboration between research groups is fast becoming the norm. Since journal publications remain the most important media for dissemination of scientific information, it is vital for researchers to have access to reputable scientific journals where they can publish their findings. Journals like *The FASEB Journal* and *FASEB BioAdvances*, which are associated with or run by scientific societies, are justifiably highly regarded by researchers in their respective disciplines. Nonetheless, with intense competition from traditional and open‐access journals, ensuring a rapid and unbiased review with constructive comments for the authors is no trivial task that our editors and staff have to handle on a daily basis.

## 8 WHAT ARE THE ADVANTAGES OF *FASEB BIOADVANCES* ?

We highlight three here. First, FASEB is a recognized brand. The organization that has been in place for over a century and represents one of the most important alliances in the biological sciences, with its broad spectrum of constituent societies. We note, however, that *FASEB BioAdvances* is a fully open‐access journal and is open to submissions from anyone, independent of a connection to the Federation and its societies. Second, there are multiple avenues for publishing. In addition to reporting new research findings, we also publish reviews on timely subjects or perspectives that make a case for a new idea or support for an old one. Articles in our Special Collections series are good examples. Finally, the journal commits to providing a fair and robust peer‐review process, rapid handling, presently averaging 22 days, and the broadest dissemination of content. *FASEB BioAdvances* eliminates as many barriers as possible so that authors are empowered to decide how they want to share their work, while also following the best practices in open‐access publishing.

